# Relative compatibility of *Schistosoma mansoni* with *Biomphalaria sudanica* and *B. pfeifferi* from Kenya as assessed by PCR amplification of the *S. mansoni ND5* gene in conjunction with traditional methods

**DOI:** 10.1186/s13071-016-1457-x

**Published:** 2016-03-21

**Authors:** Lijun Lu, Si-Ming Zhang, Martin W. Mutuku, Gerald M. Mkoji, Eric S. Loker

**Affiliations:** Center for Evolutionary and Theoretical Immunology, Department of Biology, University of New Mexico, Albuquerque, 87131 USA; Center for Biotechnology Research and Development, Kenya Medical Research Institute (KEMRI), P.O Box 54840-00200, Nairobi, Kenya; Parasitology Division, Museum of Southwestern Biology, University of New Mexico, Albuquerque, 87131 USA

**Keywords:** Schistosomiasis, *Schistosoma mansoni*, *Biomphalaria pfeifferi*, *Biomphalaria sudanica*, Compatibility, Parasite transmission, Epidemiology, Kenya

## Abstract

**Background:**

*Schistosoma mansoni* is hosted by several species of *Biomphalaria* spp. snails in Africa. We were interested in determining if there were differences in compatibility of *S. mansoni* with *Biomphalaria sudanica* from Lake Victoria, or with *B. pfeifferi* from streams and smaller water bodies in Kenya. Does this parasite develop with equal efficiency in both snail species, and does this have implications for transmission in different habitat types?

**Methods:**

Primers for PCR amplification of the *S. mansoni ND5* gene were designed and tested for sensitivity and specificity. We exposed laboratory-reared *B. sudanica* and field-derived *B. pfeifferi* to single miracidium infections and at 1, 2, 4, 8, 16 and 24 days post-exposure (dpe), snails were extracted for the PCR assay. Snails were also shed for cercariae and/or dissected prior to extraction. Additionally, *B. sudanica* and *B. pfeifferi* were collected from field locations and tested with the PCR assay.

**Results:**

The *ND5* PCR assay was sensitive (>0.1 fg *S. mansoni* genomic DNA) and allowed *S. mansoni* to be differentiated from other relevant schistosome species or snails. The number of PCR positive snails at 1–4 dpe was higher for *B. pfeifferi* than for *B. sudanica*, but not significantly so (*P* = 0.052). From 8–24 dpe, more *B. pfeifferi* harbored successfully developing parasites (positive by both dissection and PCR) than did *B. sudanica* (*P* = 0.008). At 40 dpe, more *B. pfeifferi* than *B. sudanica* shed cercariae or harbored dissection positive/PCR positive infections (*P* < 0.001). Both immature and failed (dissection negative but PCR positive) *S. mansoni* infections could also be detected in naturally infected snails of both species.

**Conclusions:**

The PCR assay detected *S. mansoni* infections in snails exposed to one miracidium for one day. Both *B. sudanica* and *B. pfeifferi* supported full development of *S. mansoni*, but *B. pfeifferi* was more compatible, with significantly more dissection positive/PCR positive or shedding infections, and significantly fewer failed infections (dissection negative/PCR positive). This highlights the relatively lower compatibility of *B. sudanica* with *S. mansoni*, and suggests the factors responsible for incompatibility and how they might affect transmission of *S. mansoni* in habitats like Lake Victoria deserve additional study.

## Background

Schistosomiasis currently infects over 230 million people [1], and relies for its transmission to humans on the production of infective cercariae that occurs in the freshwater snails that serve as intermediate hosts. Chemotherapy-based programs of schistosomiasis control have made great progress in lowering the mortality and morbidity associated with schistosome infection, but transmission control has been harder to achieve [2–4]. Control operations may fail to sufficiently diminish the input of schistosome eggs into freshwater habitats containing appropriate species of freshwater snails. Some snails consequently become infected and over a period of about one month, two generations of schistosome sporocysts are produced, culminating in the daily production of hundreds or even thousands of cercariae that may persist from a single infected snail for over a year [5]. The cercariae emerging from the population of infected snails comprise a considerable force of transmission, both to infect people not previously infected, and to effect rapid reinfection of people who may have been successfully treated in a control program.

The difficulties in preventing new infections and reinfections from occurring have reinvigorated interest in snail control [6], and provided a new impetus to better understand the biology of schistosomes in their snail hosts. For example, what factors govern how many snails become infected and how long they continue to produce cercariae? Of particular interest to this study is the question of how often schistosome infections in snails are initiated by the penetration of miracidia into snails, yet subsequently fail to undertake their full cycle of development that normally would culminate in the production of cercariae. In other words, is the force of transmission blunted significantly by a high failure rate of schistosome infections in snails? Such failure could be mediated by active defense responses by the snails [7, 8], by negative interactions with other trematode species within the intramolluscan environment [9, 10], by lack of needed nutritional or other vital components in some snails, or perhaps simply because the schistosome was damaged while penetrating the snail.

Our interest in this question was stimulated by observations made in coastal Kenya of *Bulinus nasutus* snails that were surveyed for the presence of *Schistosoma haematobium* infections [11]. It was noted that whereas only 0.14–3.4 % of the snails surveyed actually shed cercariae of *S. haematobium*, a much higher percentage of snails (28–54 %) were positive for schistosome DNA when screened using a PCR-based assay based on amplification of a repeated sequence of *S. haematobium*. This study was of particular interest because of its focus on field-derived snails in actual endemic transmission areas, and for indicating that only a small proportion of infected snails reach the stage of cercarial shedding. It suggested that interactions occurring within the snail host may significantly limit the force of transmission of infection to people.

We were interested to know if similar circumstances applied to *Schistosoma mansoni* in western Kenya where prevalence in the human population is often high, and both *Biomphalaria pfeifferi* and *B. sudanica* serve as intermediate hosts [12]. A recent study showed using both field-derived *B. pfeifferi* and *S. mansoni* eggs obtained from infected children that the prevalence of cercarial shedding obtained upon experimental infection of snails to a single miracidium ranged from about 50–70 %, suggesting compatibility in this host-parasite combination is high [5]. We sought to monitor the presence and progress of *S. mansoni* infections in both *B. pfeifferi* and *B. sudanica* using an approach similar to that employed by Hamburger et al*.* [11], albeit with a different parasite gene targeted for PCR amplification.

Several approaches have been developed for the molecular detection of *S. mansoni* in snails, including use of nested PCR amplification of the 18S ribosomal gene [13], of multi-copy repetitive elements [14], or of the mitochondrial cytochrome oxidase gene [15]. Mitochondrial minisatellite and ITS2 regions have also been amplified for sensitive *S. mansoni* detection in snails [16] and qPCR has been used to amplify the highly variable intergenic spacer region in the ribosomal gene complex and used in conjunction with fluorescent probes to identify with sensitivity and specificity particular species of *Schistosoma* in field-collected snails [17]. Loop-mediated isothermal amplification techniques have also been developed for *S. mansoni* [18, 19]. Several of these techniques have been shown to detect femtogram amounts of parasite DNA in complex backgrounds containing abundant snail DNA. These studies have been able to detect infections with a single miracidium even at short intervals (one day or less) following exposure to infection.

In this study, both the cytochrome *c* oxidase 3 (*COX3*) gene and the NADH dehydrogenase subunit 5 (*ND5*) mitochondrial gene were targeted for amplification using primers to enable specific amplification for *S. mansoni* [20]. This approach takes advantage of the multiple copies of mitochondrial DNA found within each cell, and the fact that both genes are highly variable among species of *Schistosoma*, enabling the design of species-specific primers. After deciding to focus on the *ND5* gene, we first report on the sensitivity and specificity of amplification that can be achieved using primers derived from this gene, and then further document the ability of amplification of the *ND5* gene to detect *S. mansoni* infections both in a laboratory strain of *B. sudanica* and in field-derived *B. pfeifferi* that were exposed to *S. mansoni*. We also report on its ability to detect *S. mansoni* infections in snails derived from natural habitats that were not shedding cercariae of *S. mansoni*. To go along with the PCR results, we also monitored the presence of *S. mansoni* by dissection of exposed snails and, for older infections, by examination for release of cercariae. From this study we make inferences about the compatibility of *S. mansoni* in *B. pfeifferi* and *B. sudanica* derived from west Kenya.

## Methods

### Primer design and detection

Sequence alignments were made for both the *COX3* and *ND5* mitochondrial genes from *S. mansoni*, *S. haematobium, S. japonicum, B. glabrata* and *B. sudanica.* Snails were also included because they are a potential source of cross-reacting DNA in our samples. Primers targeting unique regions of both genes from *S. mansoni* were designed using the free online software Premier primer 3.0. (http://bioinfo.ut.ee/primer3-0.4.0/). The specificity of candidate primers was tested using primer sequences in query blasts against the mitochondrial gene sequences of the other four species listed above. Three pairs of primers were designed for each gene and tested, and the primer pair Nd5-2 was selected for detection of infections because of its greater sensitivity as compared to the other candidate primers.

### Parasites and snails used

Eggs of *S. mansoni* were obtained from pooled fecal samples from school children aged 6–12 years from a primary school in Asao, Kenya (00°19'01"S, 35°00'22"E, altitude 1,171 m). Eggs were hatched using standard methods [5] and miracidia were harvested for experiments as described further below. Adult worms of *S. rodhaini* were collected and preserved in absolute ethanol at KEMRI. A Kenyan strain of *B. sudanica* originally established from snails obtained from Lake Victoria (Kisumu) was maintained at the Kenya Medical Research Institute (KEMRI). Uninfected laboratory-reared *B. sudanica* and field-derived *B. pfeifferi* from Asao stream (00°19'5.50"S, 35°0'24.99"E) were used for experimental infections. Some *B. sudanica* obtained from Lake Victoria Powerhouse (00°05'33.47"S, 34°45'11.52"E) and Carwash (00°05'45.00"S, 34°44'57.69"E) collecting sites within Kisumu (00°05'30.12"S, 34°46'4.64"E) were also used in some studies. *B. pfeifferi* were also obtained from irrigation canals at Mwea (00°49'4.80"S, 37°37'19.19"E), a temporary stream at Kasabong (00°9'6.84"S, 34°20'7.80"E) and Mangelete Canal (02°42'1.80"S, 38°05'45.30"E).

### Ethical approval

Approval was obtained from the KEMRI Ethics Review Committee (ERC) and the UNM Institutional Review Board (IRB) for all aspects of this project involving human subjects (reference number 12–182). Children were selected for the study because they are frequently infected with *S. mansoni* and are easily accessible from their schools. Prior to recruitment, the study team met with village and school officials, and parents to explain the purpose of the study. The study was explained in a language understandable by the local residents. Participation was voluntary and participants were allowed to withdraw at any time, without penalty. Written and signed consent was sought from parents/guardians, and assent acquired from children above 12 years of age. Involvement of human subjects in this project was limited to provision of fecal samples. Any child found positive for *S. mansoni* was offered standard treatment with praziquantel (40 mg/kg body weight). Children found positive for geohelminths (*Ascaris*, hookworms and *Trichuris*) were offered treatment with albendazole (500 mg) by a trained and qualified clinician. If other medical conditions were detected or suspected, the participant was referred to the nearest hospital for further medical care. To ensure confidentiality, each participant was given a personal identification number as an identifier, and all references to information/data obtained from the participant was referred to by this number. Consent forms, information and data obtained from the study participants were stored securely within KEMRI on password-protected computers.

### Experiments involving experimental exposures of snails to *S. mansoni*

Prior to testing the PCR assay with Kenyan snails, 6–9 mm laboratory-reared *Biomphalaria glabrata* (M line strain) snails were individually exposed to a single Kenyan *S. mansoni* miracidium. These snails were observed to see if miracidia penetrated or not, and DNA was then extracted from the snails and subjected to the assay, to determine if the assay results correlated well with observations of actual miracidium penetration.

Laboratory-reared *B. sudanica* (6–9 mm shell diameter) were exposed individually to one miracidium of *S. mansoni* for 16–20 h, and then transferred into aquaria for longer-term culture. Aeration was provided by plastic tubing connected to an airstone. The snails were fed on lightly boiled lettuce and water was changed in the aquaria once every week. Any snails dying post-exposure were noted and enumerated. Snails were collected and preserved in absolute ethanol at 1, 2, 4, 8, 16, and 24 days post-exposure (dpe). At 40 dpe, remaining snails were individually screened for *S. mansoni* cercariae. All snails not shedding *S. mansoni* cercariae were also preserved in absolute ethanol; most but not all were subjected to the PCR assay. Similar protocols were followed for non-shedding *B. pfeifferi* taken from Asao stream, with snails 6–9 mm in shell diameter again exposed to infection.

For snails exposed for 8 dpe or longer, prior to DNA extraction, snails were dissected and examined for the presence of *S. mansoni* sporocysts (snails with younger infections were not dissected because sporocysts are too small to readily find). DNA was extracted from the dissected snails (including fragments), as discussed further below.

### Experiments involving field snails

Some experiments to detect *S. mansoni* infections utilized *B. pfeifferi* collected from Asao Stream, Mwea, Kasabong or Mangelete Canal, or *B. sudanica* obtained from the Carwash and Powerhouse sites in Lake Victoria. Snails were collected, sorted, isolated and screened for shedding of schistosome or other trematode cercariae. Some actively shedding snails were preserved in ethanol for use in assays. Those snails not shedding were transferred into aquaria, and after 40 days, were again screened for cercariae. All of the snails that did not shed *S. mansoni* were preserved in absolute ethanol for eventual use in the PCR assay, to determine if they nonetheless harbored inapparent *S. mansoni* infections.

### DNA extraction

Preserved snails were rinsed in water to remove ethanol and in some cases were dissected to determine if larval stages of *S. mansoni* or of other trematodes were obviously present. Each snail (including all parts if dissected) was then put separately in a 1.5 mL Eppendorf tube after 600 μL CTAB solution (2 % hexadecyltrimethylammonium bromide, 100 mM Tris HCl [pH = 8.0], 20 mM EDTA, 1.4 M NaCl, 0.2 % *β*-mercaptoethanol, 0.1 mg/mL Proteinase K) [21]. Snails were crushed with a pestle, and the remains were digested with the enzyme RNAase at 60 °C for 2 h followed by phenol extraction and ethanol precipitation. The extracted DNA was suspended in TE buffer (Tris-hydrochloride buffer, pH 8.0, containing 1.0 mM EDTA) and stored at -70 °C until used.

### PCR assay

The PCR reactions were carried out using 8-tube strips with attached caps, in a volume of 20 μL containing Go-Taq Flexi DNA Polymerase (Promega, Madison, USA), buffer, and dNTPs (Promega, Madison, USA). Each reaction was performed by mixing 4 mM of MgCl_2_, 4 μL of 5× buffer, 1.0 mM of Mix dNTPs, 1U of polymerase, both forward (5’-ATT AGA GGC AAT GCG TGC TC-3’) and reverse (5’-ATT GAA CCA ACC CCA AAT CA-3’) Nd5-2 primers (20 pmol each), 2 μL of extracted and purified snail DNA, and distilled water (DNA/RNA free) up to 20 μL. The thermocycler profile was: 5 min at 94 °C, then 30 cycles each for 1 min at 95 °C, 1 min at 58 °C and 30 s at 72 °C; and a final elongation at 72 °C for 10 min. DNA from adult *S. mansoni* worms served as positive controls. Negative controls contained molecular grade water. Products were separated on 2 % agarose gels.

### DNA sequencing

Sanger sequencing was used to identify some of the amplified bands obtained. The Bigdye sequencing reactions were carried out in a volume of 10 μL using individual 0.2 mL PCR tubes, which contained 1 μL ready reaction mix (Applied Biosystems Inc.), 1.5 μL Bigdye Terminator v3.1 Sequencing buffer (5×), 1 μL forward/reverse Nd5-2 primer (2 pmol), 1–2 μL PCR clean-up product and distilled water (DNA/RNA free) up to 10 μL. The thermocylcler profile was: 1 min at 96 °C, 15 cycles each for 10 s at 96 °C, 5 s at 50 °C and 75 s at 60 °C; then 5 cycles each for 10 s at 96 °C, 5 s at 50 °C and 90 s at 60 °C; and 5 cycles each for 10 s at 96 °C, 5 s at 50 °C and 2 min at 60 °C. Sequencing products were cleaned up and submitted either to the UNM Molecular Biology Facility or to the sequencing facility of the International Livestock Research Institute in Nairobi.

### Statistical analyses

Data analysis were conducted by using RStudio© (RStudio, Inc. Boston, MA) [22] and Microsoft Excel® (Microsoft Corporation, Redmond, WA). A *P*-value < 0.05 was considered statistically significant.

## Results

### Sensitivity and specificity of the primers selected

**Fig. 1:**
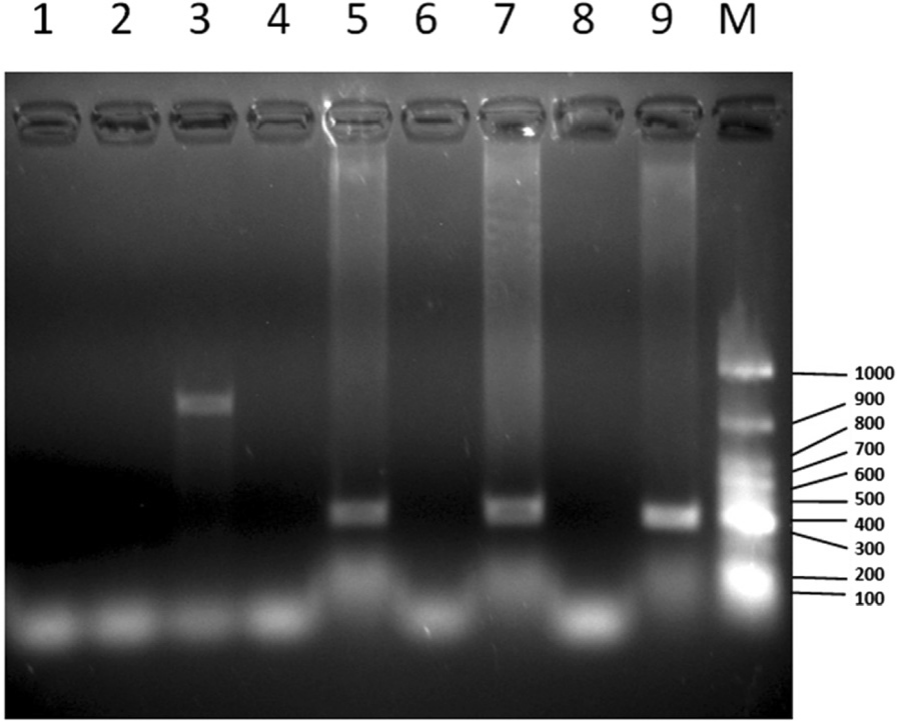
Electrophoresis of PCR amplicons obtained from different genomic DNA sources using the Nd5-2 primers. Lane 1: *S. haematobium*; Lane 2: *S. japonicum*; Lane 3: *S. rodhaini*; Lane 4: uninfected *B. pfeifferi*; Lane 5: *S. mansoni*-infected *B. pfeifferi*; Lane 6: uninfected *B. sudanica*; Lane 7: *S. mansoni*-infected *B. sudanica*; Lane 8: Negative control; Lane 9: Positive control (*S. mansoni* genomic DNA); Lane M: GeneRuler 100 bp DNA ladder (Thermo Scientific, USA). Values on the right are in base pairs

To assess their specificity, the Nd5-2 primers were tested for their ability to generate amplicons from DNA extracted from adult worms of *S. mansoni*, *S. haematobium*, *S. japonicum*, or *S. rodhaini*, from uninfected *B. pfeifferi* or *B. sudanica,* and from *B. pfeifferi* and *B. sudanica* infected with *S. mansoni*. The Nd5-2 primers amplified a 302 bp band from *S. mansoni* DNA, and from DNA from infected *B. pfeifferi* or *B. sudanica*. No bands were amplified from DNA from *S. haematobium*, *S. japonicum*, uninfected *B. pfeifferi* or uninfected *B. sudanica* (Fig. 1). The 302 bp band was verified as the expected *S. mansoni* NADH dehydrogenase subunit 5 (*ND5*) sequence (blasted in NCBI using the basic local alignment search tool, the Query cover of the alignments was 100 % and percentage identity was 99 % with an E value = 6e-129). These primers also amplified a band of ~800 bp from *S. rodhaini* DNA. This band was subsequently sequenced and also verified as bonafide *S. rodhaini* DNA (not shown).

**Fig. 2:**
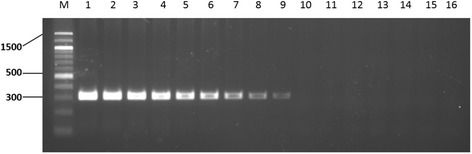
PCR amplicons resulting from different concentrations of *Schistosoma mansoni* genomic DNA using Nd5-2 primers. Ten-fold serial dilutions of genomic DNA starting from 10 ng genomic DNA (Lane 1) down to 0.0001 ag (Lane 16) were tested. Lane M: 100 bp DNA ladder (Gold Biotechnology®, USA). Values on the left are in base pairs

Sensitivity of detection of the Nd5-2 primers was tested as well (Fig. 2). DNA from *S. mansoni* adult worms was serially diluted (ten-fold) and tested, with the highest concentration tested being 10 ng (Fig. 2, Lane 1). The 302 bp bands in lane 1–9 (Fig. 2) were confirmed as the expected *S. mansoni ND5* sequence. The PCR assay detected as little as 0.1 fg *S. mansoni* genomic DNA (Fig. 2, Lane 9). The same level of sensitivity was achieved as reported using a Loop-Mediated Isothermal Amplification (LAMP) Assay [18] or oligochromatographic ‘dipstick’ technology (PCR-OC) [17].

### Experimental exposures of snails to *S. mansoni*

For 15 of 17 (88.2 %) *B. glabrata* visually confirmed to have been penetrated by a single miracidium, a positive signal was obtained in the PCR assay at 16 h post-exposure. This suggests that at this very early stage of parasite development the assay records a false negative rate of about 10 %. Consequently it might be expected that in other experiments the actual rates of infection recorded at early stages of development (1–4 dpe) may be somewhat higher than we noted based on PCR assay results alone.

**Fig. 3:**
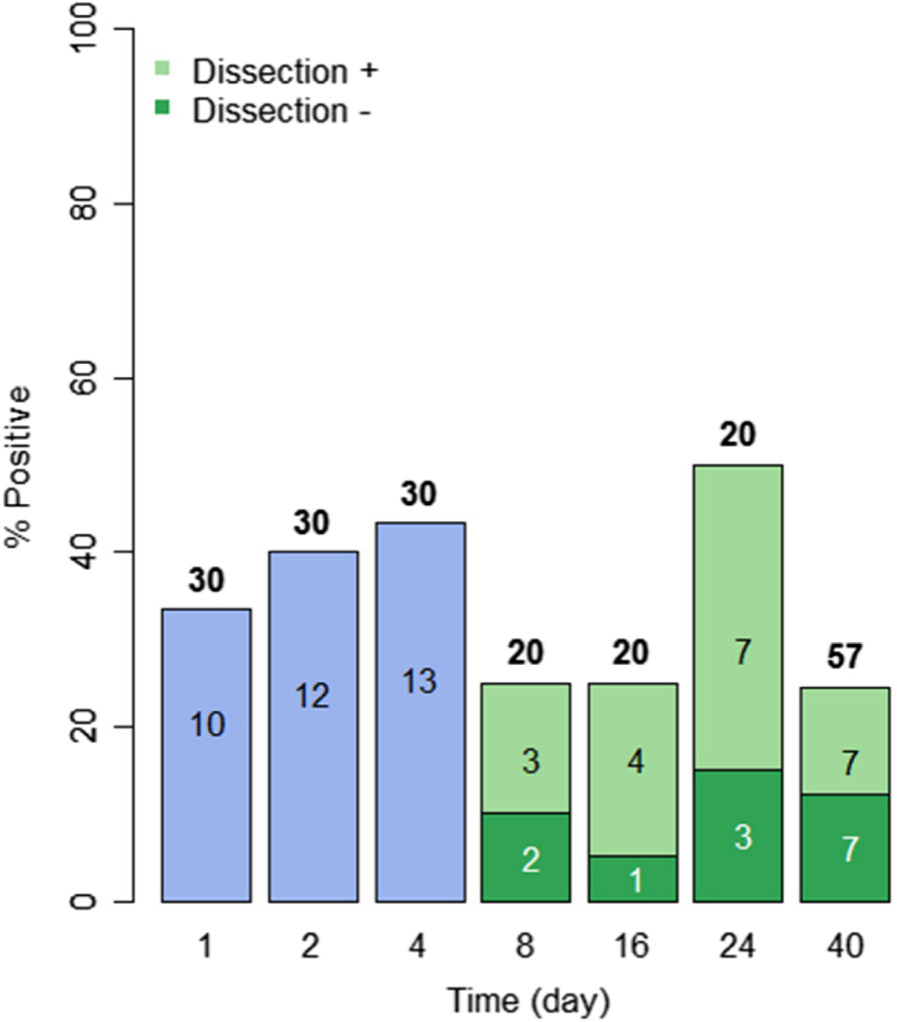
Infection status of laboratory-reared *B. sudanica* exposed individually to 1 m/s of *S. mansoni*. The number over each bar indicates the number of snails examined in the PCR assay. The number within the bar is the number of positive snails. The percentage of snails found positive in the PCR assay is indicated on the vertical axis. At 8 dpe and after, snails were also dissected prior to examination in the assay. The number of snails found to be PCR positive and dissection positive (light green) and PCR positive but dissection negative (dark green, indicative of missed or failed infections) is shown for each time point. At day 40, only non-shedding snails were examined in the assay

For *B. sudanica* individually exposed to a single miracidium of *S. mansoni*, the percentage of snails found positive at 1 dpe using the PCR assay was 33 % (Fig. 3). The percentage of PCR-positive snails did increase somewhat at both 2- and 4 dpe, again indicating the assay may miss some infections at 1 dpe. From 1–4 dpe, 38.9 % snails were PCR-positive for *S. mansoni* miracidia (Figs. 5, 6). From 8 dpe on, as sporocysts are larger and have produced germ balls by that time, it was also feasible to dissect snails to confirm microscopically if infections were present prior to extracting them for use in the PCR assay. Of 20 snails dissected at 8 dpe, 3 were shown to have sporocysts and were positive in the assay, but two additional snails were negative for visible sporocysts yet positive in the PCR assay (Fig. 3). Although the latter two snails may represent false positives, we did not encounter false positives in any of our control gel lanes. They may instead have contained infections that were too small to be seen upon dissection, simply because the sporocysts were small or possibly because they had failed to develop. Similar discrepancies, with the number of PCR-positive snails exceeding the number of dissection positive snails, were also found at 16 and 24 dpe (Fig. 3). For 8–24 dpe, of 20 snails that were PCR positive indicative of penetration, 6 (30 %) were dissection negative, suggestive of either parasites we missed or of failed infections (Figs. 5, 6).

At 40 dpe, when also taking into account the number of snails that had shed cercariae by this time, 14.7 % of the snails examined were unequivocally positive for *S. mansoni*. This included 2 of 71 (2.8 %) snails that shed cercariae and 11.9 % that were both dissection and PCR positive. Failed infections, defined as those that were dissection negative and PCR positive comprised 11.9 % of all snails; they amounted to 44.7 % of all snails that exhibited some sign of positivity to *S. mansoni* (either shedding or PCR positive). Assuming that about 38.9 % of snails were PCR positive at 1–4 dpe, then by 40 dpe the percentage of dissection-positive/PCR-positive snails together with shedding snails was only 14.7 %, with only 2.8 % shedding by that time (Figs. 3, 5, 6).

For exposures of *B. pfeifferi* to *S. mansoni* infections, the snails used were derived from the field because we lacked a laboratory colony for this species. All snails were checked prior to use and were not shedding cercariae of any kind, nor were cercariae shed by any of these snails prior to the time expected based on their deliberate exposure to *S. mansoni*. The *B. pfeifferi* snails used for this experiment were collected at a different time of year from other field snails collected, at times when preexisting natural infection rates happened to be very low.

**Fig. 4:**
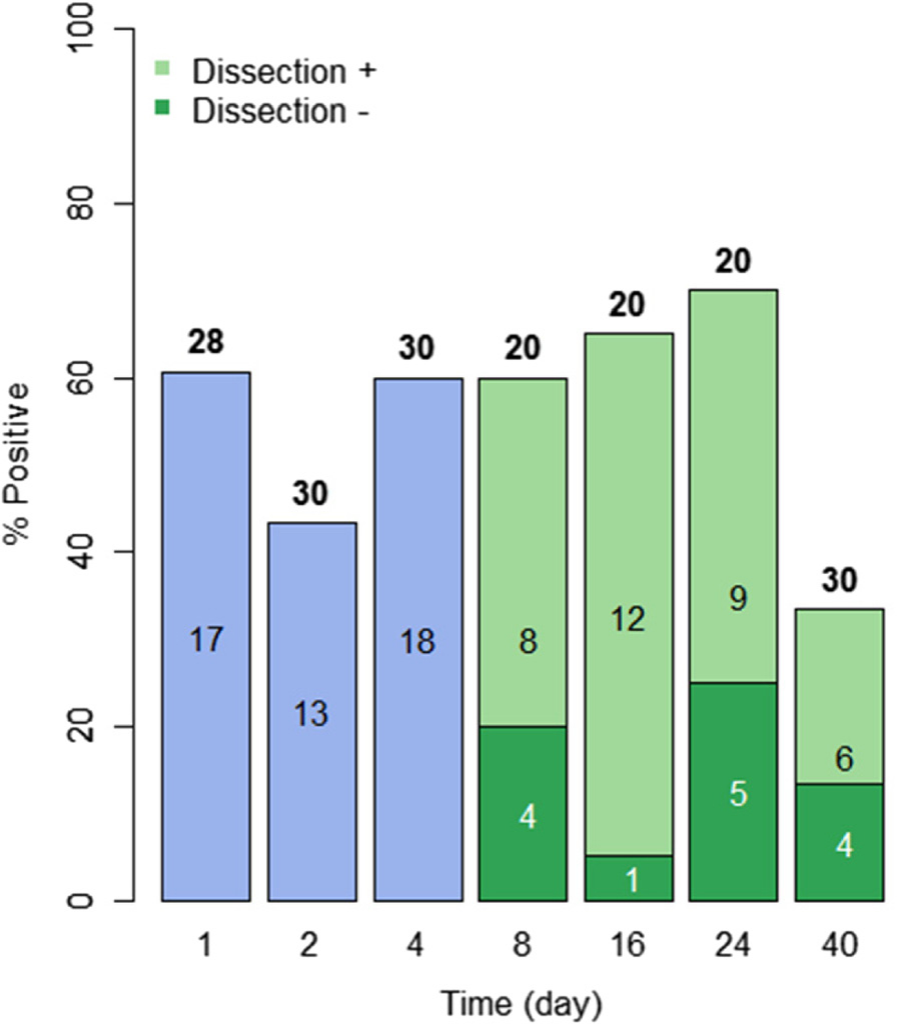
Infection status of field-derived *B. pfeifferi* exposed individually to 1 m/s of *S. mansoni*. The number over each bar indicates the number of snails examined in the PCR assay. The number within the bar is the number of positive snails. The percentage of snails found positive in the PCR assay is indicated on the vertical axis. At 8 dpe and after, snails were also dissected prior to examination in the assay. The number of snails found to be PCR positive and dissection positive (light green) and PCR positive but dissection negative (dark green, indicative of missed or failed infections) is shown for each time point. At day 40, only non-shedding snails were examined in the assay

**Fig. 5:**
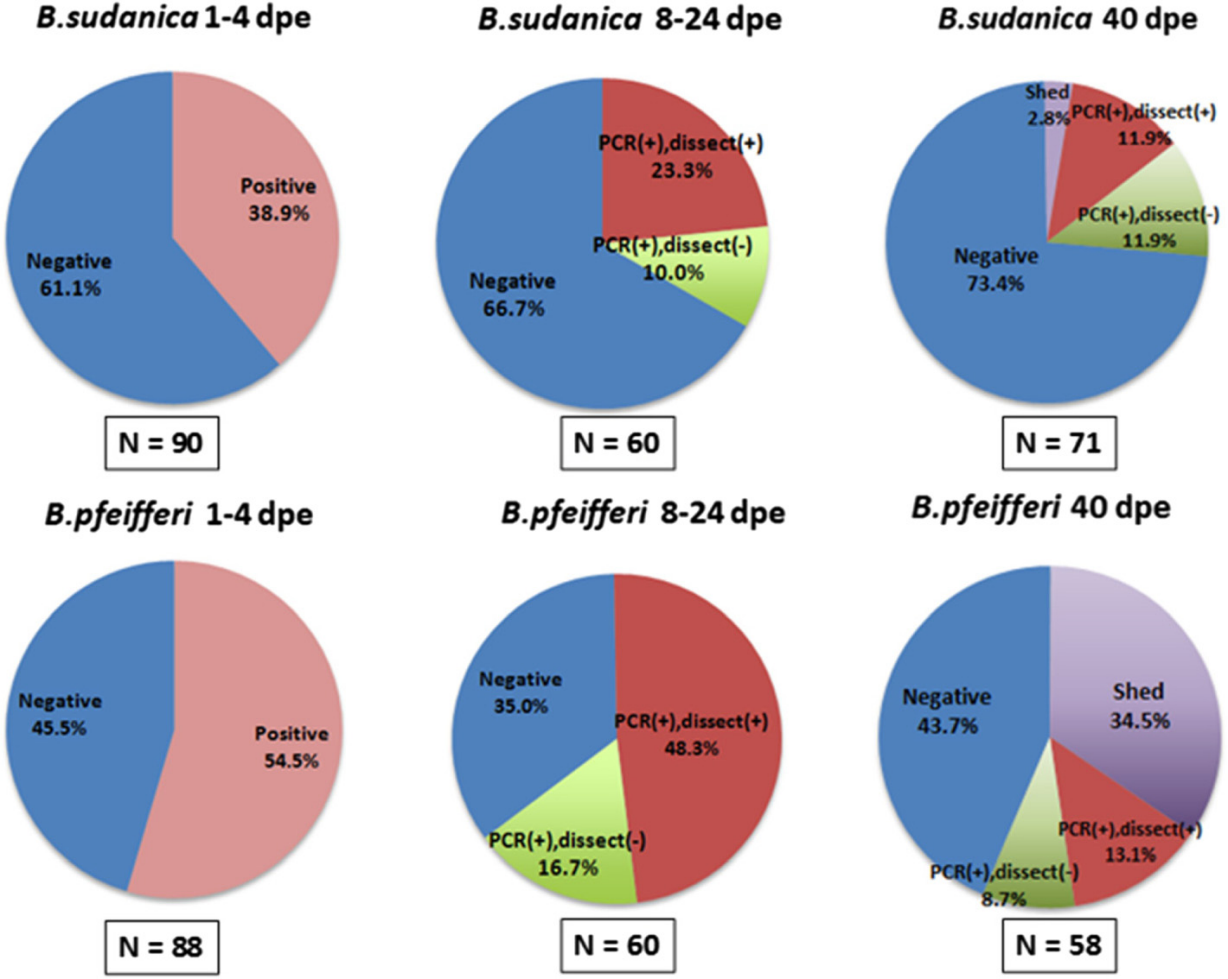
Summary of infection status for *B. sudanica* and *B. pfeifferi* at various times post-exposure. The number of snails of each species examined for each of the stipulated time intervals is shown in the box below the corresponding pie chart. Blue = negative snails; pink = PCR positive snails; red = PCR positive, dissection positive; green = PCR positive, dissection negative snails; purple = snails shedding *S. mansoni* at the 40^th^ dpe

**Fig. 6:**
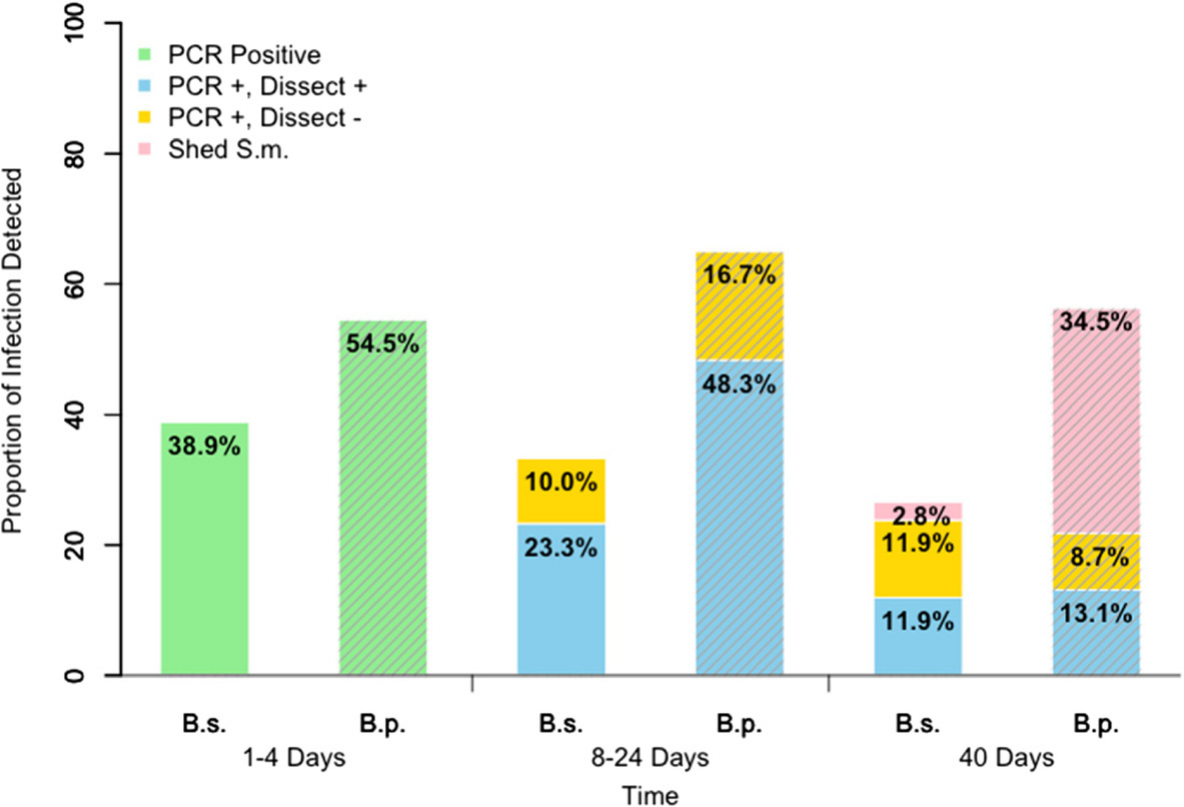
Comparison of infection status for *B. sudanica* and *B. pfeifferi* at various times post-exposure. *B. sudanica* indicated by B.s. and solid bars; *B. pfeifferi* indicated by B. p. and bars with diagonal stripes. Green = PCR positive snails at 1–4 dpe; blue = PCR positive, dissection positive snails; yellow = PCR positive, dissection negative snails; pink = snails shedding *S. mansoni* at 40 dpe. 1–4 dpe, no significant difference in *S. mansoni* infection rates (*p* = 0.052) between *B. pfeifferi* and *B. sudanica*; 8–24 dpe, significant different in successful parasite infection rates (those that are both dissection and PCR positive) between *B. pfeifferi* and *B. sudanica* (*p* = 0.008); at 40 dpe, significant difference in *S. mansoni* infection rates (including those both dissection and PCR positive, and those shedding) between two species (*p* < 0.001), including with more *B. pfeifferi* shedding cercariae than for *B. sudanica*

Over half of the snails exposed to a single miracidium were positive at 1 dpe, a percentage that dropped somewhat at 2 dpe but rose again at 4 dpe (Figs. 4, 5). Although the overall percentage of PCR-positive snails at 1–4 dpe was higher for *B. pfeifferi* than for *B. sudanica*, it did not differ significantly between snail species (Fig. 6). For 8–24 dpe, as with B*. sudanica*, more *B. pfeifferi* were PCR-positive than were found positive for sporocysts by dissection (Figs. 4, 5 and 6). Of 49 snails that were PCR positive, 25.6 % were dissection negative, suggestive of parasites we missed or failed infections.

At 40 dpe, when taking into account the number of snails that shed cercariae, 47.6 % of *B. pfeifferi* were unequivocally positive for *S. mansoni*, 34.5 % by shedding, and 13.1 % by being both dissection and PCR positive. Failed infections (dissection negative and PCR positive) comprised 8.7 % of the snails; they amounted to 15.4 % of all snails exhibiting some positivity for *S. mansoni*. Relative to a starting percentage of positive snails of 54.5 % at 1 dpe, at 40 dpe the percentage of dissection positive/PCR positive and shedding snails was 47.6 %, the latter figure significantly higher than noted for *B. sudanica* (Figs. 3, 4, 5 and 6).

Some of the snails exposed to infection died during the course of the experiment. For *B. pfeifferi*, this figure amounted to 26.9 % of all snails exposed, and for *B. sudanica* it was 7.3 %. For 40 dpe, the overall proportion of infected snails (shedding or both dissection and PCR positive) was recalculated taking into account the dead snails. Regardless of whether all dead snails were assumed to be infected or negative for *S. mansoni*, the significant interspecific difference in compatibility persisted, with *X*^2^ = 32.59 (degrees of freedom = 1, *P* = 1.14e-8) in the former case, and *X*^2^ = 14.60 (degrees of freedom = 1, *P* < 0.001) in the latter case.

For both *B. sudanica* and *B. pfeifferi*, samples from 30 snails that were PCR-positive for *S. mansoni* were sequenced and all were found to be positive for verified *S. mansoni ND5* sequences, as expected.

### Experiments involving detection of natural *S. mansoni* in field-derived snails

**Fig. 7:**
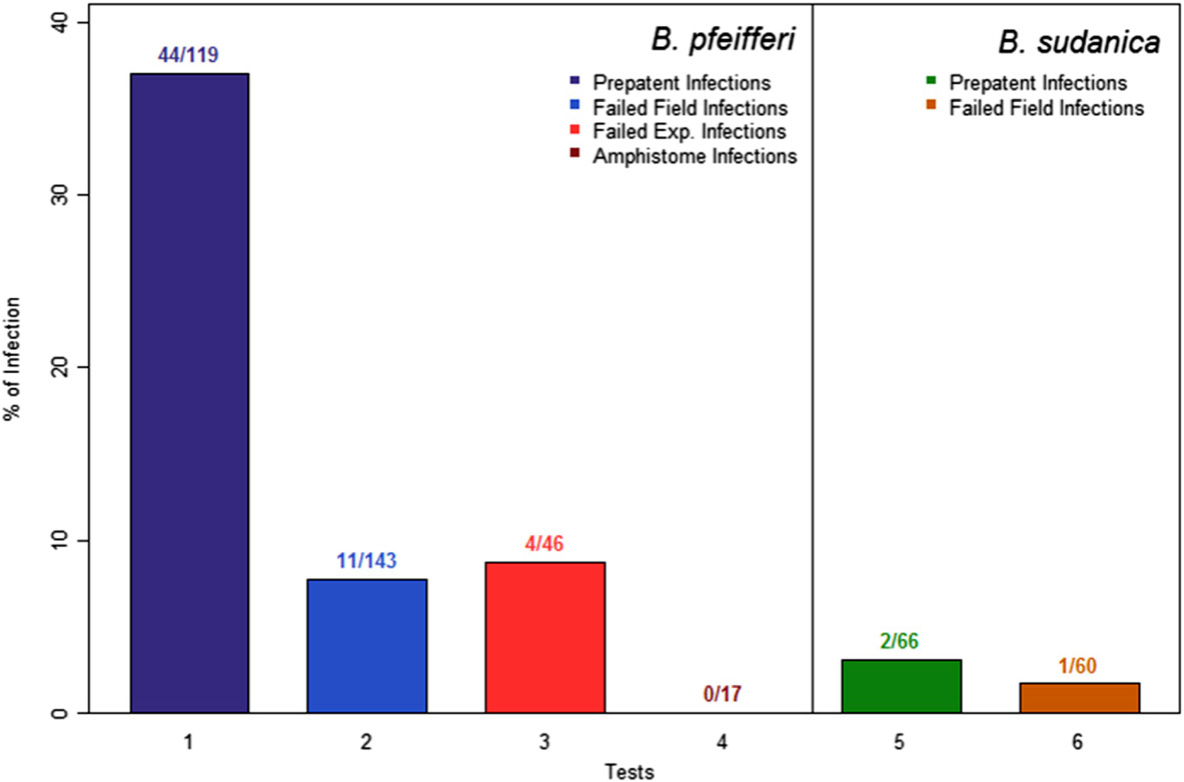
Infection status with *S. mansoni* in field-derived snails (2012–2015), using the PCR assay. 1: *B. pfeifferi* from Mwea, Mangelete Canal, Kasabong and Mwea. Snails were screened immediately after collection, and 44 of 119 snails not shedding *S. mansoni* were found positive (prepatent infections). 2: *B. pfeifferi* from Asao and Kasabong were maintained for at least 32 days after collection and all 143 snails not shedding *S. mansoni* during that interval were assayed. Those found positive (11) in the PCR assay were assumed to have failed infections. 3: *B. pfeifferi* from Mwea, 4 months after collection, non-shedders were exposed to *S. mansoni* and screened 40 days later. Non-shedders were assayed by PCR and those found positive (4) were assumed to have failed infections. 4: *B. pfeifferi* from Asao infected actively shedding amphistome cercariae, were assayed for *S. mansoni*. 5: *B. sudanica* from Carwash and Power House were screened immediately after collection, and all snails not shedding *S. mansoni* cercariae were assayed by PCR, with 2 showing prepatent infections. 6: *B. sudanica* from Carwash and Power House were maintained for 32 days and then screened. Any snails not shedding *S. mansoni* cercariae were assayed by PCR and those found positive were assumed to have failed infections

To gain a different perspective on *S. mansoni* in naturally infected field snails, we sampled *B. pfeifferi* and *B. sudanica* from different locations in Kenya from 2012 to 2015. Some *B. pfeifferi* found not to be shedding *S. mansoni* or other cercariae immediately after collection were subjected to the PCR assay (Fig. 7, column 1). Several of these non-shedding snails were nonetheless found to be PCR-positive for *S. mansoni*, which was in some cases also confirmed by dissection. This indicated the PCR assay detected naturally-acquired pre-patent infections, which proved to be surprisingly common (44 of 119, or 37 %). Other field-collected *B. pfeifferi* were held in the lab for at least 32 days post-collection (Fig. 7, column 2), and even though they never shed cercariae, and were negative upon dissection, 11 of 143 (7.7 %) were subsequently found to be PCR-positive, suggesting these snails harbored failed *S. mansoni* infections. Exposure of non-shedding field snails to *S. mansoni* (Fig. 7, column 3) also revealed failed infections (4 of 46 or 8.7 %) among those snails that subsequently failed to shed cercariae. Snails infected with amphistome rediae were checked for *S. mansoni* infections and all were PCR-negative (Fig. 7, column 4). With respect to *S. mansoni* in field-collected *B. sudanica* (Fig. 7, columns 5–6), both pre-patent infections (2 of 66 snails or 3 %) and apparent failed infections (1 of 60 snails or 1.7 %) were also detected.

For both *B. sudanica* and *B. pfeifferi*, samples from 20 field-derived snails that were PCR-positive for *S. mansoni* were sequenced and all were found to be positive for verified *S. mansoni ND5* sequences, as expected.

## Discussion

We chose to use the NADH dehydrogenase subunit 5 gene (*ND5*) of *S. mansoni* as a target for amplification, as a means for detecting infections with this parasite in its snail hosts. An increasing number of complete mitochondrial genomes for *Schistosoma* provide a good database not only for developing new perspectives for phylogenetic and population studies [20], but also for development of specific diagnostic protocols. Furthermore, mitochondrial genes offer the simple advantage of being present in multiple copies per cell. The *ND5*-based PCR assay produced amplified products from templates derived from both *S. mansoni* and *S. rodhaini*, the related *Biomphalaria*-transmitted schistosome species found in west Kenya. The two could be readily differentiated on the basis of amplicon size. Amplified products from *S. mansoni* were verified to be *ND5* by sequencing. Neither *S. haematobium* nor *S. japonicum* templates yielded amplified products. More testing with other *S. haematobium* group species, or other *Biomphalaria*-transmitted species such as *S. edwardiense* would be desirable, especially if the assay were to be used in other contexts, but these species could not have influenced the results of the present study as most of the miracidia used were from *S. mansoni* eggs from infected children. *ND5* amplicons were never retrieved from laboratory reared, schistosome-free snails (*Biomphalaria* or *Bulinus* spp.). For the purposes of this study, the technique was sensitive enough to detect one miracidium at one dpe in about 90 % of *B. glabrata* snails confirmed to have been penetrated by a miracidium. This is basically the maximum challenge required of the technique’s sensitivity. In general, the assay did not return false positive results despite repeated checking, and false PCR negatives, as from snails which we could confirm to be infected based on dissection, were also not encountered. As noted in the introduction, several molecular techniques are available to detect the presence of *S. mansoni*. Once the basic thermocycler and electrophoresis equipment are available, one sample costs $ 0.06 to prepare for a 10 μL PCR reaction, including the cost of PCR tubes, pipet tips, polymerase, primers, dNTPs and agarose. It takes 2.5 h in total to complete amplification, electrophoresis and gel documentation for 48 samples. The technique was undertaken routinely both in our labs in the U.S. and at Kisian in west Kenya.

With respect to results based on the *ND5* assay, the percentage of snails exposed to one miracidium each that were PCR positive was considerably below 100 % for both snail species, and higher for *B. pfeifferi* than for *B. sudanica*, though this was not significant (*P* = 0.052). Especially for the earliest stages of infection (1–4 dpe), we cannot rule out the possibility that there were some false negatives, but if this is so, then the proportion of PCR-positive snails might be expected to be higher at later time points when the infections were older and easier to detect. There was little evidence for this with *B. sudanica*, but perhaps some for *B. pfeifferi*. However, even with this species, the PCR-positive rates at 8–24 dpe were only slightly higher than noted at 1–4 dpe (increased from about 55 to 65 %). Other PCR-based studies to detect *S. mansoni* in snails have also recorded PCR-negative snails at one dpe to a single miracidium [13, 14]. Additionally, other studies documenting the behavior of *S. mansoni* miracidia in the presence of *Biomphalaria* snails indicate that some miracidia do not penetrate [23, 24] so it is not surprising that some snails were PCR negative in our assay, especially at the low dose used. Even in the most highly compatible combinations of *S. mansoni* and *Biomphalaria* tested [25, 26], infection rates in the 80 % range are obtained with exposures of one miracidium per snail, and in other combinations, at the same dose, infection percentages of 50–60 % are often retrieved but can certainly be much lower [25, 27]. Failure of miracidia to penetrate accounts for some of the failure to achieve 100 % infection rates. Underlying reasons for this are not clear but may involve toxic mucus factors released from the surface of snails [28], that may be of a macromolecular nature [29], and that may relate to mismatches between parasite and snail genotypes [27, 30]. For both *B. sudanica* and *B. pfeifferi*, the PCR positive rates generally held steady from 1–4 dpe, but for *B. sudanica* declined thereafter and for *B. pfeifferi* increased slightly or stayed steady thereafter. Why? In *B. sudanica*, some early *S. mansoni* infections may not have prospered and even though they were not going to yield productive infections, they could still be detected early because their DNA had not yet degraded. Certainly the precedent that some *S. mansoni* mother sporocysts either degenerate (up to 38 % observed) or are encapsulated (40 % observed) in *B. glabrata* has been established in some cases [23]. Especially for *B. sudanica* in our study, from 8 dpe on, the parasite signal in some of the snails may have been completely lost for these reasons. Dissection negative and PCR positive infections (failed infections) came to comprise 45 % of all snails exhibiting some positivity to *S. mansoni* at day 40. Our dissections may have missed some parasites, but this should be less probable in snails with older infections, assuming sporocyst development was proceeding normally. Also, at 40 dpe for *B. sudanica*, nearly half of the PCR-positive snails were also dissection positive but had not shed, suggesting that it takes longer for *S. mansoni* to develop in *B. sudanica* than in *B. pfeifferi*. Other studies have also reported slow development times for *S. mansoni* in *B. sudanica* relative to other *Biomphalaria* species [31], (Mutuku MW et al., in preparation).

As noted above, the percentage of PCR-positive snails for *B. pfeifferi* at 8–24 dpe (65 %) was somewhat higher than noted at 1–4 dpe (55 %), even though some of these (about 25 %) were apparently failed infections. By 8–24 dpe, the proportion of dissection positive/PCR positive snails was over 2× higher than for *B. sudanica*. By 40 dpe, the proportion of all snails that was unequivocally positive for *S. mansoni* was 3.2X higher and the proportion of all snails that was shedding was over 12× higher than for *B. sudanica*, also supporting the idea that *S. mansoni* develops faster in *B. pfeifferi* than in *B. sudanica*. For both snail species, it is probable that more of the dissection positive/PCR positive snails would have converted to active shedders if given more time to do so.

The mortality rate of *S. mansoni*-exposed snails was 26.9 % for *B. pfeifferi*, nearly four times higher than for *B. sudanica* (7.3 %). This is a likely consequence of the higher overall successful infection rate seen in the former species [5]. Even if all dead snails were assumed to be uninfected, *B. pfeifferi* still retained a significantly higher percentage of successful *S. mansoni* infections than did *B. sudanica*.

With respect to use of the PCR assay with field-collected snails, prepatent *S. mansoni* infections were frequently detected. The presence of *S. mansoni* in PCR-positive snails was confirmed by dissection. Prepatent rates were higher for *B. pfeifferi* than for *B. sudanica*, in general accord with results of experimental exposures, but this difference might also simply reflect differing levels of transmission at the times they were collected. Field-derived snails also yielded results suggestive of failed *S. mansoni* infections. This reinforces the concept coming from our experimental infections that some *S. mansoni* infections fail to thrive in *B. pfeifferi* or *B. sudanica*, as also noted for *B. glabrata* [23].

This study is in agreement with Frandsen [31] and Mutuku et al. [5] who found that *S. mansoni* of Kenyan origin is highly compatible with Kenyan *B. pfeifferi*, with shedding rates of over 50 % being achieved with some single miracidium infections. Frandsen’s [31] comprehensive study of several *S. mansoni* isolates from both Africa and the Americas tested against both African and American *Biomphalaria* species showed that *B. pfeifferi* was in general remarkably compatible, but that *B. sudanica* often yielded much lower infection rates, and lower cercariae production rates per snail. In some combinations, even including *S. mansoni* of African origin, *B. sudanica* proved refractory to infection. Infection rates of 0–16 % were noted for *B. sudanica* from Uganda when exposed to three miracidia per snail of local *S. mansoni* isolates, infection rates that could be raised to 41 % when higher doses of miracidia were used [32]. In a study of the ability of *B. stanleyi* (a close relative of *B. pfeifferi*) and *B. sudanica* from Lake Albert to support *S. mansoni* transmission, *B. sudanica* was shown to have lower infection rates (9.9 *vs* 21.9 %, using 4 miracidia per snail in experimental exposures), to produce fewer cercariae per day, and to shed cercariae for limited periods of time relative to *B. stanleyi* [33]. Adriko et al*.* [34] compared the compatibility of *B. pfeifferi*, *B. sudanica*, *B. choanomphala*, and *B. stanleyi* to *S. mansoni* taken from near either Lake Victoria or Lake Albert. Upon exposure to 20 miracidia per snail, they obtained infection rates ranging from 7.5–12.5 %, and did not note significant differences in compatibility between *B. sudanica* and *B. pfeifferi*, perhaps because the overall infection rates obtained for all snail species were low. They did note that *B. sudanica* produced more cercariae per day than *B. pfeifferi*.

Our study suggests that *S. mansoni* from children from Asao primary school, west Kenya is less compatible with *B. sudanica* from the Kenyan shore of Lake Victoria than with *B. pfeifferi* from nearby Asao stream. It should be noted though that L. Victoria is only 20 km from our Asao study site, and *B. sudanica* is the major snail host for *S. mansoni* along many if not most places on the Kenyan shore of the lake. Compatibility is obviously still sufficient for transmission in the lake. Mutuku et al*.* [5] showed that local adaptation effects for Kenyan *S. mansoni*, at least with *B. pfeifferi*, are not strong; high infections rates were still achieved with allopatric combinations of *B. pfeifferi* and *S. mansoni* from Kenya.

Relative to our results indicating higher compatibility with *S. mansoni* for *B. pfeifferi* than *B. sudanica*, it must be noted we examined laboratory-reared *B. sudanica* and field-derived *B. pfeifferi*. Different rearing conditions could account for the compatibility differences observed. For example, laboratory-rearing may lead to the loss of alleles at polymorphic loci that influence compatibility. It has been hypothesized that bottlenecking at such loci might strongly influence both susceptibility of strains of *B. glabrata* and infectivity of strains of *S. mansoni* [35]. Different environmental conditions in the lab and field may also have influenced relative growth rates such that even though our study exposed 6–9 mm snails of both species to infection, they were of different ages and consequently, of different susceptibilities. Yet another possibility is that field-derived *B. pfeifferi* may have had prior exposures to trematode infections (including to *S. mansoni*), and that this might have altered the susceptibility of the *B. pfeifferi* snails used relative to the laboratory-reared *B. sudanica* employed.

Although these factors may have been at play in our system, we think they are not of primary importance in dictating the compatibility difference observed. First, whereas the laboratory-associated bottlenecking effect has been invoked as something that might affect compatibility differences among strains of a single snail species, the comparison we made is an interspecific one. Definitive intrinsic differences among species of *Biomphalaria* in their compatibility with *S. mansoni* clearly exist [36, 37], regardless of whether the snails have been laboratory-reared or not. For instance, the features held by *B. glabrata* that allow this species to be in almost all cases susceptible to Neotropical *S. mansoni*, even after years of laboratory-rearing, are in contrast to those of *B. obstructa* that, despite numerous attempts, has never been shown to be capable of supporting patent *S. mansoni* infections [38]. Differences in compatibility between *B. pfeifferi* and *B. sudanica* noted above with respect to the comprehensive study of Frandsen [31] clearly persisted even though the various isolates of *S. mansoni* and snails used had been laboratory-reared for years. In a study (by Mutuku MW et al., in preparation), using standard criteria of compatibility, direct comparisons of *B. pfeifferi* and *B. sudanica* (both field-derived) exposed to *S. mansoni*, all from Kenya, retrieved the same general conclusion: *B. sudanica* is less compatible to *S. mansoni* than *B. pfeifferi*. Furthermore, even adult *B. pfeifferi* had higher infection rates with *S. mansoni* than juvenile *B. sudanica* (Mutuku MW et al., in preparation). With respect to the issue of whether field-derived *B. pfeifferi* snails may have experienced previous exposures to trematode infections that biased their compatibility to subsequent experimental exposures to *S. mansoni*, the literature would suggest that such an exposure history might favor immune priming and thus would bias against compatibility with later exposures [39]. Based on this reasoning, prior exposures might lower the compatibility of field-derived *B. pfeifferi* relative to laboratory-reared *B. sudanica*, so the high compatibility we nonetheless noted for *B. pfeifferi* may therefore represent a conservative statement of interspecific compatibility differences. For reasons stated in Adema and Loker [37], we feel it is unlikely that immune priming would be highly relevant in field populations of snails, consistent with the observation that multiple genotype infections with *S. mansoni* are common in both *B. pfeifferi* and *B. sudanica* from Kenya [40, 41]. Lastly, *S. mansoni* infections were not present or common in the pool of field-derived *B. pfeifferi* used for experimental infections as none subsequently developed patent infections ahead of schedule, indicating they had not been exposed to *S. mansoni* prior to the time of collection.

So, based on this study and in combination with results of other workers [31, 33], we believe that *B. sudanica*, at least from the Kenyan waters of Lake Victoria, may in general be less compatible with *S. mansoni* than is *B. pfeifferi*. This has some interesting potential ramifications for transmission. For instance, low compatibility and low infection rates in *B. sudanica* might simply be offset by the vast numbers of *B. sudanica* populating the shores of Lake Victoria, such that transmission still readily occurs. Transmission by *B. pfeifferi* in other locations like streams may be far more efficient on a per snail basis. Also, if compatibility of *B. sudanica* is poor, then it might be possible to exploit or embellish this trait to the detriment of *S. mansoni* transmission. Immunological studies of *B. sudanica* have not been undertaken so more work remains to be done to learn how the responses of this snail to *S. mansoni* differ from the responses of more compatible species like *B. pfeifferi* or *B. glabrata*. This in turn could yield information that helps us to understand the broader issues of why some species of *Biomphalaria* are supportive environments for *S. mansoni* whereas other species are not. This bears on the more conceptual issue of understanding the factors that govern snail host specificity for digenetic trematodes in general.

Lastly, with respect to the results of Hamburger et al*.* [11] whose results for *S. haematobium* in field-derived *Bulinus nasutus* stimulated our study, they noted that from 28–54 % of snails harbored prepatent infections, yet only 0.14–3.4 % were found to shed cercariae. This implied a 16–200 fold attrition rate for *S. haematobium* infections. In our study, the attrition from a PCR positive rate of 38.9 % for 1–4 dpe to a 2.8 % shedding rate at 40 dpe was 13.9-fold for *B. sudanica*. For *B. pfeifferi*, going from a PCR positive rate of 54.5 % at 1–4dpe to a 34.5 % shedding rate at 40 dpe represent a 1.6-fold attrition rate. By 40 dpe, failed infections among snails exhibiting some form of positivity for *S. mansoni* were about three times more likely for *B. sudanica*. We conclude that for the specific schistosome and snails populations studied, poor compatibility (whether mediated by immunological responses or lack of suitability) are unlikely to be significant impediments for *S. mansoni* transmission in *B. pfeifferi*, but may be considerably more meaningful for *B. sudanica*. This study did not examine the extent to which the onset, daily extent, and duration of cercarial shedding may also come into play in defining overall compatibility. Those studies are currently underway.

## Conclusions

We were interested in determining if two major snail vectors, *Biomphalaria sudanica* from Lake Victoria and *B. pfeifferi* from streams, differed in their compatibility with *S. mansoni* in Kenya. We developed a PCR assay to amplify the *S. mansoni ND5* gene which sensitively and specifically detected *S. mansoni* infections, even in snails exposed to one miracidium for one day. Both *B. sudanica* and *B. pfeifferi* were shown to support the full development of *S. mansoni* following single miracidium exposures, but *B. pfeifferi* was generally more compatible, with significantly more dissection positive/PCR positive or shedding infections, and fewer snails exhibiting apparent failed infections. This is in agreement with literature highlighting both the high degree of compatibility of *B. pfeifferi* and the relatively lower compatibility of *B. sudanica* with *S. mansoni*. The role of the latter species in transmission of *S. mansoni* transmission in habitats like Lake Victoria consequently deserves additional study. Further investigation of the host-related factors responsible for poor compatibility may provide novel new leads for schistosomiasis control in intractable transmission sites like Lake Victoria, and for better understanding long-standing questions like how host specificity is governed.

## Abbreviations

*B.s*: *Biomphalaria sudanica*
*B.p*: *Biomphalaria pfeifferi*
dpe: day post-exposure
KEMRI: Kenya Medical Research Institute
UNM: University of New Mexico
qPCR: quantitative Polymerase Chain Reaction
mL: milliLiter
mM: milliMole
mg: milligram
μL: microLiter
fg: femtogram
ng: nanogram
bp: base pair
